# Dr. Alexander S. Hunter

**Published:** 1896-03

**Authors:** 


Dr. Alexander S. Hunter, of New York City, died at his residence at Spuyten Duyvil, to which he had lately moved, February 13, 1896, aged 55 years. Dr. Hunter was a prominent member of the New York Academy of Medicine, of the medical society of the county of New York, and of various other medical organisations. He was an alumnus of the medical department of the University of the City of New York, class of '63, and had obtained eminence in his profession. Of late years his chief renown was in the departments of obstetrics and gynecology.

